# Swiss consensus recommendations on urinary tract infections in children

**DOI:** 10.1007/s00431-020-03714-4

**Published:** 2020-07-03

**Authors:** Michael Buettcher, Johannes Trueck, Anita Niederer-Loher, Ulrich Heininger, Philipp Agyeman, Sandra Asner, Christoph Berger, Julia Bielicki, Christian Kahlert, Lisa Kottanattu, Patrick M. Meyer Sauteur, Paolo Paioni, Klara Posfay-Barbe, Christa Relly, Nicole Ritz, Petra Zimmermann, Franziska Zucol, Rita Gobet, Sandra Shavit, Christoph Rudin, Guido Laube, Rodo von Vigier, Thomas J. Neuhaus

**Affiliations:** 1 Paediatric Infectious Diseases, Lucerne Children’s Hospital, Cantonal Hospital Lucerne, Spitalstrasse, 6000, Luzern 16, Switzerland; 2 Division of Infectious Diseases and Hospital Epidemiology, University Children’s Hospital Zurich, Steinwiesstrasse 75, 8032 Zurich, Switzerland; 3 Infectious Diseases and Hospital Epidemiology, Children’s Hospital of Eastern Switzerland, Claudiusstrasse 6, 9006 St. Gallen, Switzerland; 4 Paediatric Infectious Diseases, University of Basel Children’s Hospital, Spitalstrasse 33, 4056, Basel, Switzerland; 5 Department of Pediatric Infectious Diseases, Inselspital, Bern University Hospital, University of Bern, Freiburgstrasse 15, 3010 Bern, Switzerland; 6 Pediatric Infectious Diseases and Vaccinology Unit, Department Mother-Woman-Child, Lausanne University Hospital, Lausanne, Switzerland; 7 Pediatric Infectious Diseases, Pediatric Institute of Southern Switzerland, Ospedale Regionale di Bellinzona e Valli, Via Ospedale 12, 6500 Bellinzona, Switzerland; 8 General Pediatrics & Pediatric Infectious Diseases Unit, Department of Woman, Child and Adolescent, University Hospitals of Geneva & Medical School of Geneva, 6, rue Willy-Donzé, 1211 Geneva 14, Switzerland; 9 Department of Paediatrics, Fribourg Hospital HFR and Faculty of Science and Medicine, University of Fribourg, Fribourg, Switzerland; 10 Paediatric Infectious Diseases, Department of Paediatrics, Cantonal Hospital Winterthur, Brauerstrasse 15, 8401 Winterthur, Switzerland; 11 Paediatric Urology, University Children’s Hospital Zurich, Steinwiesstrasse 75, 8032 Zurich, Switzerland; 12 Paediatric Surgery, Lucerne Children’s Hospital, Cantonal Hospital Lucerne, Spitalstrasse, 6000 Luzern 16, Switzerland; 13 Pediatric Nephrology, University Children’s Hospital Basel, Spitalstrasse 33, CH-4031 Basel, Switzerland; 14 Pediatric Nephrology, University Children’s Hospital Zurich, Steinwiesstrasse 75, 8032 Zurich, Switzerland; 15 Pediatric Clinic, Wildermeth Children’s Hospital, Kloosweg 84, 2502 Biel-Bienne, Switzerland; 16 Paediatrics, Lucerne Children’s Hospital, Cantonal Hospital Lucerne, Spitalstrasse, 6000 Luzern 16, Switzerland

**Keywords:** Urinary tract infection, CAKUT, Vesicoureteric reflux, Guideline, Imaging, Prophylaxis

## Abstract

The kidneys and the urinary tract are a common source of infection in children of all ages, especially infants and young children. The main risk factors for sequelae after urinary tract infections (UTI) are congenital anomalies of the kidney and urinary tract (CAKUT) and bladder-bowel dysfunction. UTI should be considered in every child with fever without a source. The differentiation between upper and lower UTI is crucial for appropriate management. Method of urine collection should be based on age and risk factors. The diagnosis of UTI requires urine analysis and significant growth of a pathogen in culture. Treatment of UTI should be based on practical considerations regarding age and presentation with adjustment of the initial antimicrobial treatment according to antimicrobial sensitivity testing. All children, regardless of age, should have an ultrasound of the urinary tract performed after pyelonephritis. In general, antibiotic prophylaxis is not recommended.

*Conclusion*: Based on recent data and in line with international guidelines, multidisciplinary Swiss consensus recommendations were developed by members of Swiss pediatric infectious diseases, nephrology, and urology societies giving the clinician clear recommendations in regard to diagnosis, type and duration of therapy, antimicrobial treatment options, indication for imaging, and antibiotic prophylaxis.

**Table:** 

**What is Known:** • *Urinary tract infections (UTI) are a common and important clinical problem in childhood. Although children with pyelonephritis tend to present with fever, it can be difficult on clinical grounds to distinguish cystitis from pyelonephritis, particularly in young children less than 2 years of age.* • *Method of urine collection is based on age and risk factors. The diagnosis of UTI requires urine analysis and significant growth of a pathogen in culture.*	
**What is New:** • *Vesicoureteric reflux (VUR) remains a risk factor for UTI but* per se *is neither necessary nor sufficient for the development of renal scars. Congenital anomalies of the kidney and urinary tract (CAKUT) and bladder-bowel dysfunction play a more important role as causes of long-term sequelae. In general, antibiotic prophylaxis is not recommended.* • *A switch to oral antibiotics should be considered already in young infants. Indications for invasive imaging are more restrictive and reserved for patients with abnormal renal ultrasound, complicated UTI, and infections with pathogens other than E. coli.*

**What is Known:**

• *Urinary tract infections (UTI) are a common and important clinical problem in childhood. Although children with pyelonephritis tend to present with fever, it can be difficult on clinical grounds to distinguish cystitis from pyelonephritis, particularly in young children less than 2 years of age.*

• *Method of urine collection is based on age and risk factors. The diagnosis of UTI requires urine analysis and significant growth of a pathogen in culture.*

**What is New:**

• *Vesicoureteric reflux (VUR) remains a risk factor for UTI but* per se *is neither necessary nor sufficient for the development of renal scars. Congenital anomalies of the kidney and urinary tract (CAKUT) and bladder-bowel dysfunction play a more important role as causes of long-term sequelae. In general, antibiotic prophylaxis is not recommended.*

• *A switch to oral antibiotics should be considered already in young infants. Indications for invasive imaging are more restrictive and reserved for patients with abnormal renal ultrasound, complicated UTI, and infections with pathogens other than E. coli.*

## Introduction

The kidneys and the urinary tract are a common source of infection in children of all ages, especially infants and young children. Acute upper urinary tract infections (UTIs; pyelonephritis) may lead to substantial morbidity [1–3]. UTIs per se are only responsible for a small extent for future morbidity, e.g., arterial hypertension or chronic kidney disease [4]. The main risk factor for these sequelae is congenital anomalies of the kidney and urinary tract (CAKUT) [5]. The last Swiss consensus recommendations on UTI were published in 2013 [6]. Based on recent data and in line with international guidelines [7, 8], the Swiss recommendations have now been updated in regard to approaches to diagnosis [9–11], type and duration of antimicrobial treatment [9–14], indications for imaging [15, 16], and antibiotic prophylaxis [17–20]. As the age of the patient at presentation is an important factor regarding clinical management, specific recommendations are, where relevant, stratified by age. European guidelines on UTI management in children were published in 2015 by Stein et al. [21]. We have therefore aimed to provide up-to-date recommendations for UTI management for Switzerland but also for anyone who is managing children with UTI.

## Methods

The consensus recommendations were developed by members of the Swiss Working Group of Paediatric Nephrology (SAPN), the Pediatric Infectiology Group Switzerland (PIGS), and the Swiss Society for Paediatric Urology (SwissPU), who are experts on the management of UTI to form the recommendations committee.

The following aspects have been the focus of the current recommendations, giving added value to the clinician looking after children with suspected or confirmed UTI:

- The method of urine collection, including non-invasive methods for fast mid-stream urine sampling
- The use of ultrasound (US), voiding cystourethrogram (VCUG), and additional imaging modalities including functional MR-urography in the setting of UTI in children
- Age-appropriate indications for parenteral antibacterial treatment, including the switch to oral and total length of treatment
- The indications for antibacterial prophylaxis
- The management of risk factors including bowel dysfunction for UTI and diagnosis thereof

Based on the above aspects, the review of the literature (publications up to December 2019) was performed on PubMed, Embase, and Cochrane. The following search terms were used: UTI, cystitis, pyelonephritis, UTI and/or urine analysis and culture, catheterization, aspiration, MSU, clean catch, ultrasound, MRI, VUR, risk, CAKUT, treatment, prophylaxis, bowel-bladder dysfunction. The online search was filtered for age “birth to 18 years.” Only articles including newborns and children up to the age of 16 years were reviewed for the development of these recommendations. The following types of studies were included to answer the clinical questions: randomized controlled trials, cohort and case-control studies, and case series. If available, all of these study types were included. Data relevant to the clinical questions were extracted from the studies to answer the questions. Recommendations were then formulated. A consensus decision was adopted when evidence was low. In these cases, all relevant papers and statements were discussed by all the authors until a consensus was achieved. The individual recommendations were graded according to the level of evidence using the GRADE method [22] defining the evidence quality (high, moderate, low, or very low) and including a recommendation grade (weak or strong).

### Aim

These recommendations are aimed at health care professionals working in the hospital or out-patient setting. They give guidance on the optimal management (workup, diagnosis, treatment, and follow-up) of children with suspected and confirmed UTI as first or recurrent event. They are aimed at all age groups from neonates to adolescents up to 16 years with and without pre-existing risk factors for the development of UTI including also children with underlying CAKUT. The recommendations do not apply to children with known primary or secondary immunodeficiency or hospital-acquired UTI.

### Recommendation no. 1: Clinical suspicion of UTI

#### UTI should be considered in every child with fever without source (evidence quality: high; recommendation: strong)

For the investigation of UTI, a systematic approach is particularly important in newborns and children under 2 years presenting with fever. This age group commonly lacks typical clinical signs (see recommendation no. 2). In addition, UTI in newborns and infants can also be associated with poor feeding, failure to thrive, lethargy, or irritability, even without fever. UTI is defined as the presence of clinical signs and symptoms in combination with pyuria and significant bacteriuria. Details and special situations are described in the following recommendations.

There are a number of pre-existing factors which increase the risk of developing UTI:

- Congenital anomalies of the kidney and/or urinary tract (CAKUT), diagnosed antenatally or postnatally
- Family history of vesicoureteric reflux (VUR) or renal disease
- Uncircumcised male infants
- Abnormal urine flow or dysfunctional voiding
- Constipation
- History suggesting previous UTI or confirmed recurrent UTI

### Recommendation no. 2: Differentiation between upper and lower UTI

#### The differentiation between upper (pyelonephritis) and lower (cystitis) UTI is crucial for appropriate management (evidence quality: high; recommendation: strong)

The classic clinical signs and symptoms of UTI are pollakisuria, dysuria, loin tenderness, and fever. Infants and children who have bacteriuria and fever should be considered having acute pyelonephritis rather than cystitis. Fever may be absent in children < 2 years of age while other non-specific signs and symptoms as specified in recommendation no. 1 may be present. Hence, in children < 2 years of age, the presence of pyelonephritis should be assumed in case of doubt. Lower UTI is particularly common in girls > 2 years of age. A diagnosis of cystitis can be considered in these children when presenting with dysuria, pollakisuria, and bacteriuria; fever and loin tenderness, however, are not present (see recommendation no 4). Regarding inflammatory markers to rule in or rule out pyelonephritis, there is no robust evidence [23]. However, repeatedly low levels of inflammatory markers such as C-reactive protein (CRP < 20 mg/l) or procalcitonin (PCT < 0.5 μg/l) make the diagnosis of pyelonephritis less likely [24]. Ultrasound of the urinary tract is neither able to prove nor exclude the presence of pyelonephritis.

### Recommendation no. 3: Methods of urine collection

#### In infants and toddlers, bladder catheterization and suprapubic aspiration are recommended methods of urine collection and are considered the “gold standard” for a reliable UTI diagnosis (evidence quality: high; recommendation: strong)

Bladder catheterization is performed more frequently than suprapubic aspiration. It is considered safe, and the risk of causing an infection is low. As the catheterization of males can be difficult, it should be performed or supervised by an experienced health care professional. When considering either of these methods, pre-interventional ultrasound guidance is helpful in assessing the presence of urine in the bladder. Collection of midstream urine (MSU) is the preferred method in cooperative children with established bladder control and following appropriate instruction. A “clean catch” urine sample represents a valid alternative in infants and younger children. Here, the mid urine stream is caught by the parents or health care professional after peri-genital cleaning with sterile water or normal saline and waiting for the void. Non-invasive stimulation (bladder tapping with or without massage of the sacral area) trigger faster samples [25, 26]. The method of collection is an important factor when interpreting results from urine analysis as MSU and clean catch have a higher contamination rate compared with catheterization and suprapubic aspiration. Urine collection bags should be used only for excluding UTI. Urine from collection bags should not be sent for urine culture as the urine will usually be contaminated with perineal flora. When a collection bag is used, it is important to attach it only for a short time (15–30 min), remove it immediately after voiding, and analyze the urine without delay. In the case of pathological findings, a second urine sample should be obtained through catheterization, clean catch, or suprapubic aspiration and sent for urine culture before initiation of empiric antibiotic therapy. An overview of age-specific recommendation for urine collection is detailed in Table 1.

**Table 1:** Summary of UTI management (AST: antimicrobial susceptibility testing)

A. Procedures and investigations				
Age	Choices	Alternative		
Urine collection				
≤ 90 days	Catheterization/clean catch	Suprapubic aspiration		
> 90 days	Clean catch/catheterization	Suprapubic aspiration, collection bag (only for exclusion of UTI)		
Urine testing and culture				
≤ 90 days	Urine analysis and Culture			
> 90 days	Urine analysis	Culture if positive dipstick (Leukocyte esterase and/or nitrite) or pyuria on microscopy		
Independent of age				
Septic patient	Urine analysis and culture			
Recurrent UTI	Urine analysis and consider culture			
Clinical signs and symptoms not correlating with urine analysis results: Culture				
Additional laboratory testing (to consider)				
≤ 90 days	CRP and/or PCT, complete blood count, blood culture, plasma creatinine, sodium (Na), potassium			
> 90 days	CRP and/or PCT			
Independent of age				
Septic patient, neonates	Full sepsis workup (blood, urine and cerebrospinal fluid investigations and cultures)			
B. Empiric therapy UTI				
	≤ 30 days	31–60 days	From 61 days (> 2 months)	From 180 d (6 months)
Fever (> 38 °C) Pyelonephritis	Amoxicillin + aminoglycoside IV	Amoxicillin + ceftriaxone IV	Oral: amoxicillin-clavulanate or 3rd gen. cephalosporine	
Treatment duration	7–10 days	7–10 days	7–10 days	
Route	IV Switch to an oral antibiotic may be considered in line with AST: - If good clinical response, tolerating oral feeding - No meningitis - No sepsis at presentation - After at least 3 days iv	Start IV and switch to oral Switch to an oral antibiotic (in line with AST) if good clinical response; if sepsis at presentation consider full 7–10 days iv or may switch to oral after 3 days iv with improved general state and tolerating oral feeding	Oral Start IV (ceftriaxone) if poor general condition or unable to tolerate oral feeding	
Afebrile Cystitis				Oral: trimethoprim-sulfamethoxazole or amoxicillin-clavulanate
Treatment duration				3 days
Route				Oral
C. Reassessment after initiation of treatment				
All children should be reassessed on days 3 to 5 following UTI diagnosis for (i) clinical (and possibly laboratory) response to treatment, (ii) confirmation of the diagnosis, and (iii) possibly adaptation of the therapy according to the AST (aim: narrowing antimicrobial spectrum)				
Treatment should be ceased if the UTI diagnosis is not confirmed (in case of a negative urine culture).				
Repeat urine testing is only needed if no adequate response to treatment is seen (consider complications or other differential diagnoses).				
D. Follow-up investigations				
All children experiencing a first episode of UTI (excluding afebrile UTI in children > 180 days) should be investigated by ultrasound of the kidneys and urinary tract within 6 weeks of the diagnosis				
MCUG should only be performed in children with any of the following risk factors: CAKUT, abnormal ultrasound suggesting anatomical pathology, non-*E. coli* UTI, sepsis, inadequate response to treatment within 48 h, signs of chronic kidney disease (increased creatinine or dyselectrolytemia (sodium, potassium) or elevated blood pressure), poor urine flow, recurrent (febrile) UTIs				
E. Antibacterial prophylaxis				
Prophylaxis only to be considered in VUR grades IV and V (WHO grading I–V). If MCUG is indicated, antibiotic prophylaxis may be started and continued up to the examination.				

### Recommendation no. 4: Urine analysis and culture, additional laboratory testing

#### The diagnosis of UTI requires urine analysis and culture. Urine dipstick (leukocyte esterase and nitrite) or microscopy alone is not sufficient to definitively confirm UTI (evidence quality: high; recommendation: strong)

In the primary care setting, dipstick testing represents a fast and convenient and sufficient way to perform urine analysis. In this setting, the additional slight gain in sensitivity of microscopic analysis does not justify the added cost and time. It should be noted that in a few situations dipstick may be false negative (for example negative leucocytes and negative nitrite in children under 3 months of age who have a high voiding frequency or in infections with *Enterococcus* spp.) [27]. Dipstick may also be false positive in the context of other situations (e.g., contamination, fever due to a different cause, inflammatory processes) [28]. In the hospital laboratory, the dipstick can be combined with urine microscopy in order to slightly improve sensitivity. However, the focus here is on the gain in specificity, especially in young infants, as well as the extended microscopic assessment of urine with unclear findings on dipstick testing. Even when both tests are used, the sensitivity is not 100%, so that a urine culture is essential for a reliable diagnosis.

Indications for urine culture:

- Always in children ≤ 90 days of age with suspected UTI/fever without source
- In children > 90 days who are suspected clinically of having acute pyelonephritis and have a positive dipstick (leukocyte esterase/nitrite) and/or urine microscopy result (pyuria)
- In all children in a reduced general condition or with a high suspicion of serious bacterial illness
- In all children with recurrent UTI and underlying conditions (CAKUT, high-grade VUR (WHO grading IV and V)
- In all children if clinical symptoms and signs do not correlate with dipstick/microscopy analysis

##### Additional laboratory testing

Empiric antibacterial therapy should only be initiated after obtaining a urine sample for analysis and culture. In cases where parenteral therapy is indicated, blood cultures should always be obtained in addition to a urine culture before starting therapy. Particularly in neonates and also in infants in reduced general state, a sepsis workup (blood, urine, and cerebrospinal fluid investigations and cultures) should be obtained if possible prior to starting empiric antibacterial therapy.

An overview of age-specific recommendation for urine analysis and additional laboratory testing is detailed in Table 1.

### Recommendation no. 5: Definition of a positive urine culture

#### In urine obtained through catheterization, the growth of a single uropathogen of ≥ 10,000 CFU/ml (10^4^) and, in midstream urine samples, the growth of a single uropathogen of ≥ 100,000 CFU/ml (10^5^) are highly suggestive of UTI. (evidence quality: moderate; recommendation: weak)

In young infants (< 3 months of age) with frequent urination, growth of 1000–10,000 CFU/ml (10^3^–10^4^) in urine obtained by catheterization may already be indicative of UTI. In urine obtained by suprapubic aspiration, any bacterial growth is usually highly suggestive of UTI. In general, growth of ≥ 2 different bacterial species suggests contamination [8, 29, 30]. However, particularly in young infants, the growth of two bacterial species (particularly *E. coli* and *Enterococcus*) may be a relevant finding and should be considered to represent a true UTI if signs and symptoms and additional laboratory workup are in line with this diagnosis [31, 32]. Significant growth of so-called non-*E.coli* bacteria is frequently associated with the presence of an anatomical malformation (CAKUT) and should prompt follow-up with imaging in young children up to the age of 3 years and should also be considered in older children with incontinence or dysfunctional voiding when underlying obstipation has been ruled out [15] (see also recommendation nos. 1 and 8). In a recent meta-analysis performed by Coulthard [33] looking at the optimal bacterial colony count threshold in urine obtained from voided or invasive methods, growth of a single uropathogen at ≥ 100,000 CFU/ml (10^5^) had the highest sensitivity (0.99) for correctly diagnosing UTI independent of age and method used. On the other hand, urine culture alone should not be used as a single criterion to make the diagnosis of a UTI but should always be considered in the context of the clinical situation (pretest probability, previous history, risk factors, clinical findings, results from urine analysis, and blood examinations (see also recommendation nos. 1, 2, and 4)) to make the best possible diagnosis [34].

In rare cases, pyelonephritis may present without pyuria and/or bacteriuria: in children who present with fever without source, raised inflammatory markers, flank pain or vomiting, and normal urine analysis and bacteriology, a MRI or static isotope nephrogram may be indicated to rule out or prove the presence of (focal) pyelonephritis [35, 36].

It is not recommended in infants and children to screen for or treat asymptomatic bacteriuria. Asymptomatic bacteriuria indicates colonization of the bladder with bacteria, often non-virulent, without clinical symptoms of UTI and with a normal urine analysis [37, 38].

### Recommendation no. 6: Treatment of UTI

#### Treatment of UTI (choice of antimicrobial, route of administration) should be based on age and clinical presentation, as well as risk factors from the patients’ past medical history. In children < 60 days, consider always starting with parenteral treatment. In children > 60 days in good general condition initiating treatment orally or parenterally is equally efficacious (evidence quality: high; recommendation: strong). Local antimicrobial sensitivity patterns (if available) should be considered when choosing an empirical agent. Adjustment of the initial treatment should be done according to antimicrobial sensitivity testing (AST) of the isolated uropathogen (evidence quality: high; recommendation: strong). The clinician should choose 7 to 10 days as the total duration of antimicrobial therapy for upper UTI (evidence quality: moderate; recommendation: weak)

In general, treatment for children with suspected UTI depends on the age of the child, severity of illness, presence of concomitant gastrointestinal symptoms (e.g., vomiting), underlying medical and/or urologic comorbidities, and the local antimicrobial resistance patterns. As there is an increased incidence of urosepsis in neonates and infants aged less than 2 months, starting with parenteral antibiotic therapy is recommended. There is currently little evidence available to guide the total duration of antimicrobial therapy in children with febrile UTIs. However, treating for periods of 7–10 days is considered safe also in young children < 90 days [10–12, 39]. This duration is also considered safe, according to recent data, in parenterally treated infants < 60 days with bacteremic UTI and ruled out concomitant meningitis [11].

In children with a severe course of UTI and underlying medical and/or urological comorbidities, one may consider treating for longer periods.

### Switch to oral treatment

There is limited data regarding bioavailability of most oral antibiotics in infants below 3 months of age. Clinical and safety data in the age group < 6 months are however encouraging. A large retrospective study of infants younger than 6 months (68% were < 3 months; 19% were neonates) found no difference in treatment failure between intravenous antibiotics for 3 days or less and 4 days or more [40]. A Cochrane review (birth to 18 years included) and trial (1 to 36 months included) of acute pyelonephritis in children treated with 10–14 days of antibiotics found no difference in the duration of fever or renal damage between all intravenous antibiotics, 3 days of intravenous followed by oral antibiotics, or all oral administration [14, 41]. In a retrospective review of neonates with UTI (bacteremic and non-bacteremic included) but without meningitis, a median length of 4 days of parenteral antibiotics followed by oral treatment, no treatment failure, or relapse was observed [42].

### Summary of treatment recommendations of all age groups

1. Begin with parenteral antibiotics in children < 60 days and in children at risk of serious illness or likely unable to take oral medication (clinically unwell/septic, vomiting, poor feeding).
2. Once urine culture results and antimicrobial susceptibility testing are available, a targeted monotherapy is strongly recommended (antibiotic options and dosages are detailed in Tables 1 and 2).
3. In the case of a multidrug-resistant pathogen (e.g., extended-spectrum beta-lactamase (ESBL) producing Gram negatives), discuss infection control measures and treatment with a pediatric infectious disease specialist.
4. Do not switch to oral therapy in the event of an inadequate response to parenteral therapy, vomiting, or poor feeding.
5. Management of children for whom no standard oral antimicrobial can be identified, based on antimicrobial susceptibility testing of the urine culture, should be discussed with a pediatric infectious disease specialist.
6. In children with acute and/or chronic renal failure, with severe renal/urological malformations, neurogenic bladder, or foreign material, the optimal management strategy includes initial parenteral route of antibiotic administration; possible step down to oral medication should be determined after a multidisciplinary consensus involving nephrologists, urologists, and infectious disease specialists has been reached.
7. In case of sepsis, increasing the duration of parenteral treatment may be required.

**Table 2:** Treatment and prophylactic antimicrobial options for UTI

Antibiotic	Dosage	Maximum daily dose	Comment
A. Upper UTI (pyelonephritis): oral			
Amoxicillin/clavulanate	40 mg/kg/dose 2× daily p.o.	3 g	Based on amoxicillin component
Cefpodoxime	4 mg/kg/dose 2× daily p.o.	400 mg	Age ≥ 30 days
Cefuroxime	15 mg/kg/dose 2× daily p.o.	1 g	
Amoxicillin	40 mg/kg/dose 2× daily p.o.	3 g	
B. Upper UTI (pyelonephritis): intravenous			
Amoxicillin	25–50 mg/kg/dose 3–4× daily iv.	12 g	
Gentamicin	7.5 mg/kg/dose 1× daily i.v./i.m.		For neonates and preterms, also consult neonatal antimicrobial guidelines
Amikacin	15 mg/kg/dose 1× daily i.v./i.m.	1.5 g	For neonates and preterms, also consult neonatal antimicrobial guidelines
Tobramycin	4–6 mg/kg/dose 1× daily i.v./i.m.	7.5 mg/kg	For neonates and preterms, also consult neonatal antimicrobial guidelines
Ceftriaxone	50 mg/kg/dose 1× daily i.v./i.m.	2 g	For neonates and preterms, also consult neonatal antimicrobial guidelines
Cefuroxime	33 mg/kg/dose 3× daily i.v. / i.m.	4.5 g	
Amoxicillin/clavulanate	25–50 mg/kg/dose 3–4× daily iv.	12 g	Based on amoxicillin component
C. Lower UTI (cystitis): oral			
Trimethoprim-sulfamethoxazole	3–5 mg/kg/dose 2× daily p.o.	320 mg	Based on trimethoprim; age > 30 days; contraindicated in hyperbilirubinaemia
Amoxicillin	25 mg/kg/dose 2× daily p.o.	4 g	
Amoxicillin/clavulanate	25 mg/kg/dose 2× daily p.o.	4 g	Based on amoxicillin
Cefuroxime	10–15 mg/kg/dose 2× daily p.o.	1 g	
D. Antibiotic prophylaxis			
Trimethoprim	1.5 mg/kg/dose 2× daily or 2 mg/kg/dose 1× daily (evening)	320 mg	Neonates and children: twice daily regimen for children still wearing diapers; Infectotrimet® suspension can be ordered by local pharmacy from neighboring countries. No Swissmedic application necessary.
Trimethoprim-sulfametoxazole	1 mg/kg/dose 2× daily or 2 mg/kg/dose 1× daily (evening)	320 mg	Age > 30 days; dose based on trimethoprim (TMP); twice daily regimen for children still wearing diapers.
Nitrofurantoin	1 mg/kg/dose 2× daily or 2 mg/kg/dose 1× daily (evening)	100 mg/dose	Age > 30 days
Amoxicillin	10 mg/kg/dose 2× daily		Use for prophylaxis only for neonates (if trimethoprim not available)

Age-specific recommendations for empiric antibiotic therapy and duration are detailed in Tables 1 and 2. Options for empiric oral agents in Switzerland are currently amoxicillin-clavulanate or 3rd generation cephalosporin. Deciding which of these two options is the first-line empiric oral agent should be done locally. AST patterns by pathogen, age, in- vs. out-patient, and area of living in Switzerland are published online (www.anresis.ch) and can help with the above decision. Antibiotic dosages and maximal daily dosages are, where available, in accordance with SwissPedDose (www.swisspeddose.ch).

### Recommendation no. 7: Follow-up during the course of infection

#### On days 3 to 5 after initial diagnosis and start of empirical antibiotic therapy, children should be clinically reviewed to assess the response to treatment and confirming the diagnosis. Urine culture results should be reviewed, and medication adjusted (narrowing the antimicrobial spectrum) if indicated based on the AST. If there is no significant growth from urine, the empirical antimicrobial therapy should be stopped, and an alternative diagnosis evaluated. (evidence quality: high; recommendation: strong)

A repeated evaluation of the child after day 3 is only necessary if there are signs of ongoing or worsening infection, i.e., if the child is still febrile or the clinical condition has not improved. In these cases, the initial working diagnosis may have to be reconsidered and other differential diagnoses or complications such as a pyonephrosis or (peri)renal abscess evaluated. Performance of an ultrasound of kidneys and urinary tract in these situations is necessary **(**Table 1**).**

### Recommendation no 8: The role and timing of urinary tract imaging in pyelonephritis

#### All children, regardless of age, should have an ultrasound of the urinary tract performed after the first episode of pyelonephritis (evidence quality: moderate; recommendation: weak). Micturition cystourethrogram should only be planned under certain circumstances. (evidence quality: moderate; recommendation: weak)

Ultrasound of the kidneys and urinary tract during the acute phase can neither rule in nor rule out an upper UTI (pyelonephritis) or VUR. It may detect anatomical malformations or the rare case of pyonephrosis (pus in the urinary collecting system: pyelon and/or ureter) with secondary obstruction. In children with typical upper UTI (pyelonephritis) responding to treatment, ultrasound, performed in the acute phase, does not influence management. It is recommended to perform an ultrasound during the acute infection to identify structural anomalies in the following situations [8, 43–45]:

- Children presenting with features of atypical upper UTI (sepsis or septic shock, poor urine flow, abdominal or bladder mass, increased creatinine, failure to respond to treatment with suitable antibiotics within 48 h)
- Children presenting with recurrent UTI

If the above features and aspects are not present, ultrasound can be performed at a later stage after pyelonephritis.

In children with non*-E. coli* UTI responding well to antibiotics and with no other features of atypical infection (as stated above), the ultrasound can be performed after the acute phase (Table 1).

#### Micturition cystourethrogram

The aim of micturition cystourethrogram (MCUG) is to detect:

- High-grade VUR (WHO grades IV and V)
- Posterior urethral valves (PUV) in boys
- Bladder and ureteric anomalies (for example ureterocele)

We recommend performing MCUG only in the following circumstances:

- CAKUT and/or dilatation of the urinary tract on ultrasound (an isolated mild dilatation of the renal pelvis (≤ 10 mm) is not an indication for further imaging)
- Poor urine flow, e.g., posterior urethral valves [PUV] in boys, oliguria not due to dehydration, urinary retention
- Infection with organisms other than *E. coli*
- Failure to respond to treatment with suitable antibiotics within 48 h
- Increased creatinine (according to age) or abnormal electrolytes (for example, hyponatremia and hyperkalemia with suspicion of secondary transient pseudohypoaldosteronism) or arterial hypertension
- Recurrent pyelonephritis (2 or more episodes)

MCUG should not be performed in the first episode of UTI in a neonate or young infant as routine examination if above-listed circumstances are not present [15, 46]. If there is a family history of VUR (WHO grades IV and V), a MCUG may be considered on an individual basis. MCUG is indicated mainly in the setting of UTI for children up to the age of 3 years as it is rare that a child first presents with signs and symptoms or complications of VUR, PUV, or bladder abnormalities at a later age [7]. In older children, bowel-bladder dysfunction and constipation are more likely responsible for first and particular recurrent UTI and should therefore be screened for (see recommendation 10).

#### Timing of MCUG

If MCUG is indicated after UTI, it may be performed as soon as available by the local radiology department. The early timing of MCUG (within 8 days after onset of antimicrobial therapy) does not influence the detection of VUR as shown in a recent review [47].

#### Antibiotic prophylaxis and MCUG

If MCUG is indicated, antibiotic prophylaxis may be started and continued up to the time of the examination (see Table 2 for options).

#### Further imaging modalities

If a pediatric radiology department has experience in contrast-enhanced ultrasound, this method (without exposing the child to ionizing radiation) can be used as an alternative to MCUG primarily in girls. In boys, it cannot rule out posterior urethral valves (PUV). Depending on findings on ultrasound and MCUG and after interdisciplinary (pediatric nephrology/urology) discussion, functional imaging (MR urography, scintigraphy) may be performed depending on the local expertise of the radiology department and availability of pediatric anesthesiology support.

### Recommendation 9: Indications for long-term antibiotic prophylaxis

#### In general, antibiotic prophylaxis is not recommended (evidence quality: high; recommendation: strong)

In the following circumstances, antibiotic prophylaxis may be indicated (planned duration should be documented):

- Children with complex CAKUT or with underlying bladder dysfunction (only after interdisciplinary—pediatric nephrology/urology/infectious diseases—review)
- Children with high-grade VUR (WHO grades IV and V)*
- If MCUG is indicated, antibiotic prophylaxis may be started and continued until the time of the examination

*In children with VUR grade III, prophylaxis should be discussed on an individual basis with parents. The number needed to treat (NNT) for prophylaxis are 5500 antibiotic doses to prevent one UTI [48]. Antibiotic prophylaxis has not been shown to reduce renal scarring [17, 18, 49]. Side effects of antibiotics, contribution to resistance problems [48], and influence on the intestinal microbiome should also be considered [50]*.*

There are no evidence-based guidelines on the duration of antibiotic prophylaxis. The indication should be reviewed after 6–12 months in correlation with clinical course and imaging follow-up. A second MCUG is generally not recommended and should always be preceded by an interdisciplinary discussion by the involved specialists.

#### Choice of antibiotic for prophylaxis

Trimethoprim (if available as a single substance) is a suitable prophylactic option in neonates. To prevent the development of resistance, beta-lactam and quinolone antibiotics should not be used. Newborns are an exception, where amoxicillin is an accepted prophylactic agent. The prophylactic antimicrobial should not be chosen based on the AST of the urine culture. If two or more UTIs develop under running prophylaxis, consider changing prophylaxis to a different agent. Options and dosages are detailed in Table 2**.**

Antimicrobial dosages and maximal daily dosages are, where available, in accordance with SwissPedDose recommendations (www.swisspeddose.ch).

### Recommendation 10: Bladder-bowel dysfunction is a relevant risk factor for recurrent UTI (evidence quality: high; recommendation: strong)

Lower urinary tract dysfunction, e.g., dysfunctional voiding during daytime and lack of complete bladder emptying (residual urine) in combination with bowel dysfunction, e.g., constipation, are labeled as bladder-bowel dysfunction (BBD). BBD is a relevant risk factor for recurrent UTI. Workup of these risk factors includes bladder/bowel diaries, questionnaires, or behavioral and psychological screening. Furthermore, it is advisable to perform uroflowmetry if dysfunctional voiding is suspected. It may be combined with transcutaneous electromyography (EMG) of the perineal muscles, particularly when uroflowmetry shows staccato voiding to differentiate the underlying cause more precisely [51]. The advantage of combining EMG with uroflowmetry is the ability to reveal intermittent contractions of the peri-urethral striated or levator ani muscles during voiding. This may aid in the preparation of pelvic floor muscle training (biofeedback) or neuromodulation with the help of urophysiotherapy [52]. Dysfunctional voiding can cause high intra-vesical pressure and lack of complete bladder emptying predisposing to secondary VUR. A further and common risk factor is chronic constipation which should be evaluated and treated promptly in children with recurrent UTI with or without VUR [44].

### Recommendation 11: Surgical and endoscopic intervention should be considered in selected cases on an individual basis (evidence quality: moderate; recommendation: weak)

In children with high-grade VUR with recurrent infections on prophylactic antibiotics or parental hesitancy to use antibiotics, surgical intervention (e.g., endoscopic injections of bulking agents or ureteric reimplantation) may be an alternative especially after the first year of life. There is no consensus about the timing and type of surgical correction [53].

## Abbreviations

ASB: Asymptomatic bacteriuria
AST: Antimicrobial sensitivity testing
CAKUT: Congenital anomalies of the kidney and urinary tract
CFU: Colony forming units
CRP: C-reactive protein
ESBL: Extended-spectrum beta-lactamase
MCUG: Micturition cystourethrogram
MDR: Multi drug resistant
MSU: Midstream urine
PCT: Procalcitonin
UTI: Urinary tract infection
VUR: Vesicoureteric reflux
