# Correction to: Swiss consensus recommendations on urinary tract infections in children

**DOI:** 10.1007/s00431-020-03820-3

**Published:** 2020-10-01

**Authors:** Michael Buettcher, Johannes Trueck, Anita Niederer-Loher, Ulrich Heininger, Philipp Agyeman, Sandra Asner, Christoph Berger, Julia Bielicki, Christian Kahlert, Lisa Kottanattu, Patrick M. Meyer Sauteur, Paolo Paioni, Klara Posfay-Barbe, Christa Relly, Nicole Ritz, Petra Zimmermann, Franziska Zucol, Rita Gobet, Sandra Shavit, Christoph Rudin, Guido Laube, Rodo von Vigier, Thomas J. Neuhaus

**Affiliations:** 1 Paediatric Infectious Diseases, Lucerne Children’s Hospital, Cantonal Hospital Lucerne, Spitalstrasse, 6000 Luzern 16, Switzerland; 2 Division of Infectious Diseases and Hospital Epidemiology, University Children’s Hospital Zurich, Steinwiesstrasse 75, 8032 Zurich, Switzerland; 3 Infectious Diseases and Hospital Epidemiology, Children’s Hospital of Eastern Switzerland, Claudiusstrasse 6, 9006 St. Gallen, Switzerland; 4 Paediatric Infectious Diseases, University of Basel Children’s Hospital, Spitalstrasse 33, 4056 Basel, Switzerland; 5 Department of Pediatric Infectious Diseases, Inselspital, Bern University Hospital, University of Bern, Freiburgstrasse 15, 3010 Bern, Switzerland; 6 Pediatric Infectious Diseases and Vaccinology Unit, Department Mother-Woman-Child, Lausanne University Hospital, Lausanne, Switzerland; 7 Pediatric Infectious Diseases, Pediatric Institute of Southern Switzerland, Ospedale Regionale di Bellinzona e Valli, Via Ospedale 12, 6500 Bellinzona, Switzerland; 8 General Pediatrics & Pediatric Infectious Diseases Unit, Department of Woman, Child and Adolescent, University Hospitals of Geneva & Medical School of Geneva, 6, rue Willy-Donzé, 1211, Geneva 14, Switzerland; 9 Department of Paediatrics, Fribourg Hospital HFR and Faculty of Science and Medicine, University of Fribourg, Fribourg, Switzerland; 10 Paediatric Infectious Diseases, Department of Paediatrics, Cantonal Hospital Winterthur, Brauerstrasse 15, 8401 Winterthur, Switzerland; 11 Paediatric Urology, University Children’s Hospital Zurich, Steinwiesstrasse 75, 8032 Zurich, Switzerland; 12 Paediatric Surgery, Lucerne Children’s Hospital, Cantonal Hospital Lucerne, Spitalstrasse, 6000 Luzern 16, Switzerland; 13 Pediatric Nephrology, University Children’s Hospital Basel, Spitalstrasse 33, CH-4031 Basel, Switzerland; 14 Pediatric Nephrology, University Children’s Hospital Zurich, Steinwiesstrasse 75, 8032 Zurich, Switzerland; 15 Pediatric Clinic, Wildermeth Children’s Hospital, Kloosweg 84, 2502 Biel-Bienne, Switzerland; 16 Paediatrics, Lucerne Children’s Hospital, Cantonal Hospital Lucerne, Spitalstrasse, 6000 Luzern 16, Switzerland

**Correction to: European Journal of Pediatrics**

10.1007/s00431-020-03714-4

The article “Swiss consensus recommendations on urinary tract infections in children”, written by Michael Buettcher, Johannes Trueck, Anita Niederer-Loher, Ulrich Heininger, Philipp Agyeman, Sandra Asner, Christoph Berger, Julia Bielicki, Christian Kahlert, Lisa Kottanattu, Patrick M. Meyer Sauteur, Paolo Paioni, Klara Posfay-Barbe, Christa Relly, Nicole Ritz, Petra Zimmermann, Franziska Zucol, Rita Gobet, Sandra Shavit, Christoph Rudin, Guido Laube, Rodo von Vigier, Thomas J. Neuhaus, was originally published electronically on the publisher’s internet portal on 03 July 2020 without open access.

With the author(s)’ decision to opt for Open Choice the copyright of the article changed on 22 September 2020 to © The Author(s) 2020 and the article is forthwith distributed under a Creative Commons Attribution 4.0 International License, which permits use, sharing, adaptation, distribution and reproduction in any medium or format, as long as you give appropriate credit to the original author(s) and the source, provide a link to the Creative Commons licence, and indicate if changes were made. The images or other third party material in this article are included in the article’s Creative Commons licence, unless indicated otherwise in a credit line to the material. If material is not included in the article’s Creative Commons licence and your intended use is not permitted by statutory regulation or exceeds the permitted use, you will need to obtain permission directly from the copyright holder. To view a copy of this licence, visit http://creativecommons.org/licenses/by/4.0.

